# Genetic landscape of Parkinson’s disease and related diseases in Luxembourg

**DOI:** 10.3389/fnagi.2023.1282174

**Published:** 2023-12-20

**Authors:** Zied Landoulsi, Sinthuja Pachchek, Dheeraj Reddy Bobbili, Lukas Pavelka, Patrick May, Rejko Krüger, the NCER-PD Consortium

**Affiliations:** ^1^LCSB, Luxembourg Centre for Systems Biomedicine, University of Luxembourg, Esch-sur-Alzette, Luxembourg; ^2^Parkinson Research Clinic, Centre Hospitalier de Luxembourg (CHL), Luxembourg, Luxembourg; ^3^Transversal Translational Medicine, Luxembourg Institute of Health (LIH), Strassen, Luxembourg

**Keywords:** Parkinson’s disease, genetics, Luxembourg, polygenic risk score, copy number variants

## Abstract

**Objectives:**

To explore the genetic architecture of PD in the Luxembourg Parkinson’s Study including cohorts of healthy people and patients with Parkinson’s disease (PD) and atypical parkinsonism (AP).

**Methods:**

809 healthy controls, 680 PD and 103 AP were genotyped using the Neurochip array. We screened and validated rare single nucleotide variants (SNVs) and copy number variants (CNVs) within seven PD-causing genes (*LRRK2*, *SNCA*, *VPS35*, *PRKN*, *PARK7*, *PINK1* and *ATP13A2*). Polygenic risk scores (PRSs) were generated using the latest genome-wide association study for PD. We then estimated the role of common variants in PD risk by applying gene-set-specific PRSs.

**Results:**

We identified 60 rare SNVs in seven PD-causing genes, nine of which were pathogenic in *LRRK2*, *PINK1* and *PRKN*. Eleven rare CNVs were detected in *PRKN* including seven duplications and four deletions. The majority of *PRKN* SNVs and CNVs carriers were heterozygous and not differentially distributed between cases and controls. The PRSs were significantly associated with PD and identified specific molecular pathways related to protein metabolism and signal transduction as drivers of PD risk.

**Conclusion:**

We performed a comprehensive genetic characterization of the deep-phenotyped individuals of the Luxembourgish Parkinson’s Study. Heterozygous SNVs and CNVs in *PRKN* were not associated with higher PD risk. In particular, we reported novel digenic variants in PD related genes and rare *LRRK2* SNVs in AP patients. Our findings will help future studies to unravel the genetic complexity of PD.

## Introduction

Parkinson’s disease (PD) is the fastest growing neurodegenerative disorder, affecting more than 8.5 million people (Dorsey and Bloem, 2018). The main pathological hallmarks of PD include loss of dopaminergic neurons in the substantia nigra and the presence of intraneural Lewy bodies, with motor and non-motor symptoms (Bloem et al., 2021). The etiology of sporadic PD is complex and influenced by both environmental and genetic factors. Familial monogenic forms defined by rare and pathogenic variants in autosomal dominant (e.g., *SNCA*, *LRRK2*, *VPS35*) or recessive (*PRKN*, *PINK1*, *PARK7*) PD-related genes, account for less than 10% of Mendelian cases (Lesage and Brice, 2009; Karimi-Moghadam et al., 2018). The contribution of genetics in the remaining patients with sporadic forms of PD is not yet well defined. Common variants have also been described as a risk factor for PD (Nalls et al., 2019). The presence of heterozygous variants in the *GBA1* gene has emerged as a common risk factor for PD, estimated to occur in about 4–12% of PD patients (Pachchek et al., 2023). The major haplotype (H1) of the microtubule-associated protein Tau (*MAPT*) gene has been also associated with increased risk of PD (Skipper et al., 2004; Zabetian et al., 2007). Additionally, a specific *MAPT* H1 sub-haplotype (H1c) has been strongly linked with progressive supranuclear palsy (PSP; Myers et al., 2005). Disease susceptibility may be influenced by a combined effect of more than 90 common low-risk genetic loci defined by large genome-wide association studies (Nalls et al., 2019; Blauwendraat et al., 2020), including those in the *SNCA* and *LRRK2* genes. Although less explored than common single-nucleotide variants (SNVs), copy number variants (CNVs) have been reported, especially in PD-associated genes where pathogenic deletions and duplications have been identified using either a gene candidate approach (*PRKN*, *SNCA*, *PINK1*, *PARK7* and *ATP13A2*) (Toft and Ross, 2010; Pankratz et al., 2011; La Cognata et al., 2017) or genome-wide burden analysis (Liu et al., 2013; Sarihan et al., 2021).

Despite ongoing global scientific efforts in genetic analysis, improvements are still needed in terms of early diagnosis and prognosis, causative treatments, and new therapeutic approaches. As the population ages, the number of PD patients will increase dramatically. It is therefore important to generate reliable evidence on the epidemiology and genetic etiology of PD to enable precision medicine and prevention for neurodegeneration in PD. In particular, three genetic discoveries that have led to new therapeutic approaches (targeting alpha-synuclein, glucocerebrosidase and *LRRK2* pathway) are now in clinical development (Sardi et al., 2018).

We had previously performed a comprehensive screening of *GBA1* gene variants (Pachchek et al., 2023). Here, we sought to genetically characterize patients with PD or atypical parkinsonism (AP) in the Luxembourg Parkinson’s Study screening for rare SNVs, CNVs, and estimated the effect of common SNVs using polygenic risk scores (PRS).

## Materials and methods

### Cohort characteristics

A total of 1,592 individuals (783 cases and 809 neurologically healthy controls) were recruited from March 2015 to December 2022 as part of the Luxembourg Parkinson’s Study, a large longitudinal monocentric study within the framework of the NCER-PD [National Centre for Excellence in Research in PD (Hipp et al., 2018; Pavelka et al., 2022)]. The most up-to-date diagnostic status of the participants was used at the time of export (July 2023). Assignment of diagnosis was based on the following diagnostic criteria: for PD UKPDSBB (Litvan et al., 2003); for progressive supranuclear palsy (PSP) Institute of Neurological Disorders and Stroke/Society criteria (Litvan et al., 1996); for frontotemporal dementia with parkinsonism (FTD-P) (Neary et al., 1998); for multiple system atrophy (MSA) (Gilman et al., 2008); for dementia with Lewy bodies (DLB) (McKeith et al., 2005). Of these individuals, 680 fulfilled the criteria for PD and 103 for AP (52 for PSP, 26 for LBD, 14 for MSA, 10 for corticobasal syndrome (CBS), and one for FTD-P). All subjects gave written informed consent. The study was approved by the National Research Ethics Committee (CNER Ref: 201407/13).

### Genotyping and quality controls

Samples were genotyped with the customized NeuroChip array, which was designed to contain tagging rare and common variants associated with neurodegenerative diseases [v.1.0 and v1.1; Illumina, San Diego, CA (Blauwendraat et al., 2017)]. These disease-targeted variants include loci from the largest completed meta-analysis of PD cases and controls, which identified many of the known PD mutations and additional rare high-risk variants. Using PLINK v1.9 (Chang et al., 2015), we performed two rounds of quality control (QC). The first round included the following steps: samples with call rates <95%, missingness rates >5%, Hardy–Weinberg equilibrium *p*-value <1e-6 and whose genetically determined sex deviated from the sex reported in clinical data were excluded from the analysis. We also removed samples exhibiting excess heterozygosity (F statistic >0.2). After these steps, the remaining samples were used for rare variant screening and validation process. Next, we performed a second round of QC steps where the filtered variants were checked for relatedness [using KING (Manichaikul et al., 2010)] and samples with first-degree relatedness were excluded. To determine the genetic ancestry, we calculated the first 10 principal components (PCs) using PLINK and merged our data with the 1,000 genomes dataset. We selected only samples of European ancestrys, excluding those with a value >3SD based on the first and the second PCs. Samples passing QC were then imputed using the Haplotype Reference Consortium r1.1 2016 on a local instance of the Michigan Imputation Server (Das et al., 2016) and filtered for imputation quality (*R*2 > 0.3). Imputed genetic variants passing QC numbered 19,490,906 SNPs.

### *MAPT* haplotype association

Using the imputed NeuroChip genotyping data, we selected rs1800547 to differentiate the *MAPT* H1 and H2 haplotypes and six tagging variants (rs1467967, rs242557, rs3785883, rs2471738, rs8070723, and rs7521) to define the H1 specific sub-haplotypes (Pittman, 2005). H1c sub-haplotype is defined by the following alleles (rs1467967(A), rs242557(A), rs3785883(G), rs2471738(T), rs8070723(A) and rs7521(G)). The association between *MAPT* haplotypes genotypic and allelic frequency in cases and controls was tested using the Chi-squared and Fisher’s exact tests, respectively. *P* values were considered significant at *p* < 0.05.

### Variant annotation and rare variant screening

We annotated the variants with ANNOVAR [v 2020-06-08 (Wang et al., 2010)]. We searched for variants within a list of “PD genes” including the major causal genes according to MDS gene classification^1^ (1) Dominant forms of classical parkinsonism, *LRRK2*, *SNCA*, *VPS35*, (2) Recessive forms of early-onset parkinsonism: *PRKN*, *PARK7*, and *PINK1*; (3) Atypical parkinsonism: *ATP13A2*. We then selected rare nonsynonymous and splicing (+/−2 bp) rare variants based on the minor alleles frequency (MAF) <1% in the Genome Aggregation Database [gnomAD r2.1 (Karczewski et al., 2020)] exomes and genomes for the non-Finnish European (NFE) population. We performed Sanger sequencing to confirm all rare variants within these PD genes. The pathogenicity of the validated rare variants was assigned based on ClinVar (Landrum et al., 2014), the MDSgenes pathogenicity score (see text footnote 1), the Combined Annotation Dependent Depletion (CADD) (Rentzsch et al., 2019) and the Rare Exome Variant Ensemble Learner REVEL scores (Ioannidis et al., 2016). CADD provides ranking scores that predict the deleteriousness of variants, considering conservation and functional information, and variants with scores equal to or greater than a CADD score of 20 are in the 1% most deleterious. REVEL is an ensemble method for predicting the pathogenicity of missense variants by integrating multiple scores. Scores range from 0 to 1 and variants with higher scores are more likely to be pathogenic. Scores greater than 0.5 are predicted to be ‘likely disease causing’, as 75.4% of known disease mutations but only 10.9% of neutral variants have a score greater than 0.5 (Ioannidis et al., 2016).

### Copy number variant calling

We generated a custom population B-allele frequency (BAF) and GC wave-adjusted log R ratio (LRR) intensity file using GenomeStudio (v2.0.5 Illumina) for all the samples that passed genotyping QC steps and used PennCNV (v1.0.5, Wang et al., 2007) to detect CNVs. Only autosomal CNV were targeted for CNV calling, as calls from sex chromosomes are often of poor quality. Adjacent CNV calls were merged into one single call if the number of overlapping markers between them was less than 20% of the total number when the two segments were combined. We conducted an intensity-based QC to exclude samples with low-quality data. After intensity-based QC, all samples had an LRR standard deviation <0.25, an absolute value of the waviness factor < 0.05 and a BAF drift <0.01. Spurious CNV calls in known problematic genomic regions (provided by PennCNV) were also removed prior the analysis. We excluded additional samples with a total number of CNVs calls greater than 80 (this threshold corresponds to the median + 3 SDs of the total number of CNVs per sample). Called CNVs were removed from the dataset if they spanned <20 SNPs, were < 20 kilobases (kb) in length and had a SNP density < 0.0001 (number of markers/length of CNVs). Additionally, SNP density was not considered for CNVs spanning ≥20 SNPs and ≥ 1 Mb in length. CNVs were then annotated for gene content using refGene including gene name and the corresponding exonic coordinates in the hg19 assembly using ANNOVAR (v 2020-06-08). We then searched for CNVs in the same list of “PD genes” used to screen for rare SNVs. We assessed the frequency of CNVs based on complete overlap with CNVs of the same copy number reported in gnomAD-SV (Collins et al., 2020) and in the Database of Genomic Variants (DGV) (MacDonald et al., 2014). We evaluated the clinical impact of the detected CNVs using the CNV-ClinViewer (Macnee et al., 2023), which integrates clinical interpretation of CNVs according to the ACMG guideline and the ClassifyCNV scores. Selected CNVs were validated using the multiplex ligation-dependent probe amplification (MLPA) assay.

### Polygenic risk score calculation

PRSs were calculated for healthy controls and PD cases using the R package PRSice2 (Choi and O’Reilly, 2019) with default parameters. PRSs for each sample were generated using the imputed genotyping data from the Luxembourg Parkinson’s Study and the summary statistics of 90 genome-wide significant SNVs that were previously reported to be associated with PD risk in the largest PD genome-wide association study (GWAS) statistics to date (Nalls et al., 2019). PRSice2 implements the clumping and thresholding method. The criteria for linkage disequilibrium (LD) clumping of SNPs were pairwise LD *r*2 < 0.1 within a 250 kb window. PRSs were computed at different GWAS *p*-value thresholds (from 5e-08 to 5e-01). PRSice2 identified the best *p*-value threshold for selecting variants that explained the maximum variance in the target sample. The predictive accuracy of the PRS model was determined by two methods: by the observed phenotypic variance (PRS model fit, R2) calculated by PRSice2 and by the area under the receiver operating curve (AUC, pROC R package). The phenotypic variance R2 was adjusted for a PD prevalence of 0.005 (Bandres-Ciga et al., 2020). The PRS distributions between healthy controls and PD cases were compared using the Wilcoxon rank-sum test. PRSs for curated gene-sets were generated using the *msigdb* function implemented in PRSice2, based on a collection of 3,090 canonical pathways from the molecular signature database (MSigDB^2^, “c2.cp.v2023.1.Hs.symbols.gmt”) with an MAF threshold of 0.01. The summary statistics of PD GWAS from Nalls et al. (2019) (excluding 23&me data) were used as the base dataset. The mapping file “Homo_sapiens.GRCh37.87.gtf” was used as the universal background for gene-set analysis. Resulting gene-sets with a value of *p* less than 0.05, corrected for Bonferroni multiple testing, were considered significant. In order to understand which biological processes were associated with PD after excluding known risk factors, we performed the same analysis after removing the 90 PD GWAS hits (Nalls et al., 2019) and additional SNVs that were located 1 Mb upstream and downstream. We used a logistic regression model to calculate the odds ratio (OR) to assess whether PRS could predict PD risk. Age, sex and the first five PCs were included as covariates.

## Results

### Cohort description

After the quality control procedure, the final dataset for the Luxembourg Parkinson’s study comprised 1,490 individuals (667 PD cases, 99 atypical PD cases and 724 healthy controls). Detailed demographic data are summarized in Table 1. The control group had a mean age at assessment of 65.8 ± 11.6 years. The PD patients had a mean age of onset (AAO) of 62.3 ± 11.8 years. To illustrate the ethnic composition of our cohort, we performed PCA using 1,000 Genome populations as a reference (The 1000 Genomes Project Consortium, 2012) and showed that all our samples clustered strongly with the European ancestry (Supplementary Figure 1).

**Table 1:** Demographic data of cases and healthy controls from the Luxembourg Parkinson’s study.

	*N*	Sex (% male)	Age at assessment (mean ± SD)	Age at onset (mean ± SD)	Family history of PD (n 1st degree relative, %)
Controls	724	54.2	65.8 ± 11.6		132 (18.2%)
PD	667	68.0	73.0 ± 11.0	62.3 ± 11.8	97 (14.5%)
PSP	50	62.0	76.4 ± 6.7	67.6 ± 7.4	5 (10%)
LBD	25	68.0	77.8 ± 9.8	70.5 ± 9.4	3 (12%)
MSA	13	69.2	75.2 ± 7.7	65.8 ± 8.4	1 (7.7%)
CBS	10	30.0	77.1 ± 7.8	69.2 ± 8.6	1 (10%)
FTD-P	1	0	69	58	0 (0%)

Counts, means and percentage were calculated after genotyping data quality controls. PD, Parkinson’s disease; PSP, progressive supranuclear palsy; LBD, Lewy body dementia, MSA, Multiple System Atrophy; CBS, corticobasal syndrome; FTD-P, Fronto-temporal dementia with parkinsonism.

### Rare variants in PD-related genes

We screened for rare (gnomAD NFE MAF < 1%) exonic and splice region variants in seven PD causal genes and validated these findings by Sanger sequencing. We identified 60 rare variants (59 missense and one frameshift) in all PD-related genes except for *SNCA* (Table 2), in 119 individuals including 52 controls, 57 PD and 10 AP patients, representing 7.9% of the total cohort (Table 3). All carriers were heterozygous, except two PD patients that were homozygous for *LRRK2* p.I723V and *PINK1* p.L369P, respectively.

**Table 2:** Rare single nucleotide variants in PD related genes in the Luxembourg Parkinson’s study.

Gene	c.DNA	Protein	CADD	REVEL	ClinVar prediction for PD	MDSgene pathogenicity	rsid
*ATP13A2*	c.C35T	p.T12M	21.9	0.497	*NA*	*NA*	rs151117874
	c.C3170T	p.A1057V	25.8	0.07	*NA*	*NA*	rs201610681
	c.G515A	p.R172H	31	0.781	*NA*	*NA*	rs776601823
	c.C746T	p.A249V	10.24	0.473	*NA*	*NA*	rs145515028
	c.A829T	p.S277C	24.2	0.729	*NA*	*NA*	rs538497077
	c.C1073T	p.P358L	23	0.555	*NA*	*NA*	rs757503427
	c.G1229A	p.R410Q	23	0.312	*NA*	*NA*	rs190746040
	c.C1294G	p.L432V	14.16	0.24	*NA*	*NA*	rs149372969
	c.G2771A	p.R924H	32	0.967	*NA*	*NA*	rs564643512
	c.A2836T	p.I946F	22.5	0.292	*NA*	*NA*	rs55708915
	c.G2939A	p.R980H	21.2	0.63	*NA*	*NA*	rs150748722
	c.A3361T	p.T1121S	10.04	0.149	*NA*	*NA*	rs41273151
*LRRK2*	c.A382G	p.S128G	20.4	0.044	Uncertain significance	*NA*	rs187299177
	c.A784G	p.M262V	0.001	0.013	Uncertain significance	*NA*	rs182233369
	c.C856G	p.L286V	23	0.173	Uncertain significance	*NA*	rs200437744
	c.G1000A	p.E334K	22.4	0.194	Conflicting interpretations of pathogenicity	*NA*	rs78501232
	c.A1004G	p.N335S	10.47	0.053	*NA*	*NA*	rs989570613
	c.G1108A	p.A370T	25.7	0.186	*NA*	*NA*	rs200189771
	c.A2167G	p.I723V	12.73	0.045	Benign	*NA*	rs10878307
	c.G2291A	p.S764N	8.77	0.01	*NA*	*NA*	rs774818561
	c.G2378T	p.R793M	23.5	0.305	Conflicting interpretations of pathogenicity	*NA*	rs35173587
	c.C2594T	p.S865F	23.1	0.149	Benign	*NA*	rs142700458
	c.G2769C	p.Q923H	14.21	0.262	Conflicting interpretations of pathogenicity	*NA*	rs58559150
	c.G2918A	p.S973N	23.4	0.13	*NA*	*NA*	rs75148313
	c.G3451A	p.A1151T	20.4	0.029	Uncertain significance	*NA*	rs74985840
	c.T3477G	p.S1159R	20.7	0.165	*NA*	*NA*	rs200965490
	c.G3683C	p.S1228T	16.12	0.307	Uncertain significance	*NA*	rs60185966
	c.G3974A	p.R1325Q	32	0.553	Conflicting interpretations of pathogenicity	*NA*	rs72546338
	c.C4321T	p.R1441C	23.3	0.727	Pathogenic	pathogenic	rs33939927
	c.C4321A	p.R1441S	22.6	0.66	Pathogenic	*NA*	rs33939927
	c.G4541A	p.R1514Q	22.3	0.1	Benign	*NA*	rs35507033
	c.T4939A	p.S1647T	13.97	0.086	Benign	*NA*	rs11564148
	c.T5606C	p.M1869T	22.8	0.514	Conflicting interpretations of pathogenicity	*NA*	rs35602796
	c.G5822A	p.R1941H	23	0.24	Uncertain significance	*NA*	rs77428810
	c.G6055A	p.G2019S	31	0.97	Pathogenic	pathogenic	rs34637584
	c.G6688A	p.E2230K	20.7	0.049	Uncertain significance	*NA*	rs201317931
	c.C7067T	p.T2356I	19.59	0.154	Conflicting interpretations of pathogenicity	*NA*	rs113511708
	c.T7169C	p.V2390A	14.66	0.165	Uncertain significance	*NA*	rs376230626
	c.G7183A	p.E2395K	23.1	0.168	*NA*	*NA*	rs78964014
	c.G7224A	p.M2408I	14.27	0.055	*NA*	*NA*	rs60545352
	c.G7483A	p.V2495I	16.34	0.022	Benign	*NA*	rs150062967
*PARK7*	c.G310T	p.A104S	25.7	0.72	*NA*	*NA*	rs774005786
	c.G535A	p.A179T	16.87	0.127	Uncertain significance	*NA*	rs71653622
*PINK1*	c.A377G	p.Q126R	14.44	0.353	*NA*	*NA*	rs775809722
	c.G836A	p.R279H	26	0.522	Uncertain significance	Possibly pathogenic	rs74315358
	c.A952T	p.M318L	23.9	0.596	Uncertain significance	*NA*	rs139226733
	c.G1015A	p.A339T	23.5	0.386	Conflicting interpretations of pathogenicity	Probably pathogenic	rs55831733
	c.T1106C	p.L369P	26.5	0.83	*NA*	Probably pathogenic	rs1195888869
	c.G1147A	p.A383T	13.23	0.405	Conflicting interpretations of pathogenicity	*NA*	rs45515602
	c.G1231A	p.G411S	20.7	0.429	Conflicting interpretations of pathogenicity	*NA*	rs45478900
	c.G1426A	p.E476K	14.31	0.127	Benign	Benign	rs115477764
	c.G1609A	p.A537T	24.1	0.297	Uncertain significance	*NA*	rs771032673
*PRKN*	c.101_102del	p.Q34fs	*NA*	*NA*	Pathogenic	*NA*	*NA*
	c.C245A	p.A82E	0.765	0.559	Conflicting interpretations of pathogenicity	*NA*	rs55774500
	c.G500A	p.S167N	15.67	0.164	Benign	*NA*	rs1801474
	c.A574C	p.M192L	22.5	0.519	Benign	*NA*	rs9456735
	c.C766T	p.R256C	32	0.811	Uncertain significance	Probably pathogenic	rs150562946
	c.C823T	p.R275W	29.5	0.747	Pathogenic	Definitely pathogenic	rs34424986
*VPS35*	c.A110G	p.N37S	21.1	0.054	Uncertain significance	*NA*	rs777006799
	c.G151A	p.G51S	22.6	0.236	Benign	*NA*	rs193077277

Rare single nucleotide variants (SNVs) were screened from Neurochip array and validated by Sanger sequencing. We included ClinVar prediction only for Parkinson’s disease (benign includes both ‘benign’ and ‘likely benign’ prediction, Pathogenic includes both ‘pathogenic’ and ‘likely pathogenic’ prediction). CADD, Combined Annotation Dependent Depletion. REVEL, Rare Exome Variant Ensemble Learner.

**Table 3:** Number and phenotypes of rare variant carriers in PD related genes.

n PD	n atypical parkinsonism	n Controls	*LRRK2*	*VPS35*	*PRKN*	*PINK1*	*PARK7*	*ATP13A2*
2	0	0	p.A1151T	*NA*	*NA*	*NA*	*NA*	*NA*
0	1 PSP	0	p.A370T	*NA*	*NA*	*NA*	*NA*	*NA*
0	0	1	p.E2230K	*NA*	*NA*	*NA*	*NA*	*NA*
0	0	2	p.E334K	*NA*	*NA*	*NA*	*NA*	*NA*
1	0	0	p.G2019S	*NA*	p.A82E	*NA*	*NA*	*NA*
3	0	1	p.G2019S	*NA*	*NA*	*NA*	*NA*	*NA*
1	0	0	p.G2019S, p.Q923H	*NA*	*NA*	*NA*	*NA*	*NA*
4	1 CBS	1	p.I723V*	*NA*	*NA*	*NA*	*NA*	*NA*
1	0	0	p.L286V	*NA*	*NA*	*NA*	*NA*	*NA*
0	0	2	p.M1869T	*NA*	*NA*	*NA*	*NA*	*NA*
0	0	2	p.E2395K	*NA*	*NA*	*NA*	*NA*	*NA*
1	0	0	p.M2408I	*NA*	p.R256C	*NA*	*NA*	*NA*
0	1 PSP	0	p.M262V	*NA*	*NA*	p.A383T	*NA*	*NA*
1	0	0	p.N335S	*NA*	*NA*	*NA*	*NA*	*NA*
1	0	2	p.R1325Q	*NA*	*NA*	*NA*	*NA*	*NA*
1	0	1	p.R1441C	*NA*	*NA*	*NA*	*NA*	*NA*
1	0	0	p.R1441S	*NA*	p.S167N	*NA*	*NA*	*NA*
1	0	0	p.R1514Q	p.N37S	*NA*	*NA*	*NA*	*NA*
5	1 CBS	2	p.R1514Q	*NA*	*NA*	*NA*	*NA*	*NA*
1	0	1	p.R1941H	*NA*	*NA*	*NA*	*NA*	*NA*
0	0	1	p.R793M	*NA*	*NA*	*NA*	*NA*	*NA*
1	0	0	p.S1159R	*NA*	*NA*	*NA*	*NA*	*NA*
0	0	1	p.S1228T	*NA*	*NA*	*NA*	*NA*	*NA*
0	0	1	p.S128G	*NA*	*NA*	*NA*	*NA*	*NA*
3	0	0	p.S1647T	*NA*	*NA*	*NA*	*NA*	*NA*
1	0	0	p.S764N	*NA*	*NA*	*NA*	*NA*	*NA*
1	0	0	p.S865F	*NA*	*NA*	*NA*	*NA*	*NA*
1	0	0	p.S973N	*NA*	*NA*	*NA*	*NA*	*NA*
0	1 DLB	3	p.T2356I	*NA*	*NA*	*NA*	*NA*	*NA*
1	0	0	p.V2390A	*NA*	*NA*	*NA*	*NA*	*NA*
0	0	1	p.V2495I	*NA*	*NA*	*NA*	*NA*	*NA*
0	0	1	*NA*	*NA*	p.R256C	*NA*	*NA*	p.R172H
1	0	0	*NA*	*NA*	*NA*	*NA*	*NA*	p.A1057V
1	0	2	*NA*	*NA*	*NA*	*NA*	*NA*	p.A249V
2	0	2	*NA*	*NA*	*NA*	*NA*	*NA*	p.I946F
1	0	2	*NA*	*NA*	*NA*	*NA*	*NA*	p.L432V
0	0	1	*NA*	*NA*	*NA*	*NA*	*NA*	p.P358L
1	0	0	*NA*	*NA*	*NA*	*NA*	*NA*	p.R410Q
1	0	0	*NA*	*NA*	*NA*	*NA*	*NA*	p.R924H
0	0	1	*NA*	*NA*	*NA*	*NA*	*NA*	p.R980H
0	0	1	*NA*	*NA*	*NA*	*NA*	*NA*	p.S277C
1	0	0	*NA*	*NA*	*NA*	p.A339T	*NA*	p.T1121S
0	0	2	*NA*	*NA*	*NA*	*NA*	*NA*	p.T12M
1	0	1	*NA*	*NA*	*NA*	*NA*	p.A104S	*NA*
1	0	1	*NA*	*NA*	*NA*	*NA*	p.A179T	*NA*
1	0	2	*NA*	*NA*	*NA*	p.A339T	*NA*	*NA*
2	0	1	*NA*	*NA*	*NA*	p.A383T	*NA*	*NA*
1	1 DLB	0	*NA*	*NA*	*NA*	p.A537T	*NA*	*NA*
1	0	0	*NA*	*NA*	*NA*	p.E476K	*NA*	*NA*
0	1 PSP / 1 DLB	0	*NA*	*NA*	*NA*	p.G411S	*NA*	*NA*
1	0	0	*NA*	*NA*	*NA*	p.L369P*	*NA*	*NA*
0	0	1	*NA*	*NA*	p.A82E	p.M318L	*NA*	*NA*
1	1 DLB	2	*NA*	*NA*	*NA*	p.M318L	*NA*	*NA*
1	0	0	*NA*	*NA*	*NA*	p.Q126R	*NA*	*NA*
0	0	1	*NA*	*NA*	*NA*	p.R279H	*NA*	*NA*
0	0	2	*NA*	*NA*	p.R256C	*NA*	*NA*	*NA*
3	0	1	*NA*	*NA*	p.A82E	*NA*	*NA*	*NA*
0	0	1	*NA*	*NA*	p.M192L	*NA*	*NA*	*NA*
1	0	1	*NA*	*NA*	p.Q34fs	*NA*	*NA*	*NA*
2	1 PSP	2	*NA*	*NA*	p.R275W	*NA*	*NA*	*NA*
0	0	1	*NA*	*NA*	p.S167N	*NA*	*NA*	*NA*
1	0	1	*NA*	p.G51S	*NA*	*NA*	*NA*	*NA*

* homozygous carriers, One PD patient was homozygous for *LRRK2* p.I723V and one for *PINK1* p.L369P.

Among the 29 rare variants identified in *LRRK2*, five variants have a CADD score > 20 and REVEL score > 0.5 (p.R1325Q, p.R1441S, p.R1441C, p.M1869T, and p.G2019S) showing high evidence for pathogenicity. Three of these variants were classified as pathogenic for PD in ClinVar (Table 2) and were present in nine individuals representing 0.60% of the total cohort (Table 3). Among these variants, five PD patients carried the extensively studied pathogenic variant p.G2019S while two PD patients carried the pathogenic p.R1441C and p.R1441S variant.

Two control individuals with family history of PD (Table 3) had rare *LRRK2* variants. One control individual carried the variant p.G2019S (38 years old) and has a high probability of developing PD (Healy et al., 2008). Another control individual (77 years old) carried the p.R1441C, although this variant is described as highly penetrant [more than 90% of carriers had PD by the age of 75 (Haugarvoll and Wszolek, 2009)].

In the autosomal recessive PD-causing genes (*PRKN*, *PARK7*, *PINK1*, and *ATP13A2*), we identified 28 heterozygous rare variant carriers and only one homozygous rare variant carrier (*PINK1* p.L369P, Table 2). The distribution of these variants was similar between cases and controls (27 PD, six AP and 29 controls, value of *p* = 0.39, Table 3). Four controls and 10 patients had a first-degree family-history of PD. The age of the control individuals carrying these heterozygous variants ranged from 52 to 85 years (mean = 67.8 years). The AAO of the PD patients carrying these heterozygous variants ranged from 39 to 87 (mean = 65.5). One PD patients was younger than 40 years (carrying *PINK1* p.A383T), all the others were older than 50 years.

According to ClinVar, two pathogenic *PRKN* variants (p.R275W and p.Q34fs) were found in three PD patients, one PSP patient and three controls (all heterozygous, representing 0.46% of the total cohort, Table 3). Three *PRKN* variants (p.M192L, p.R256C and p.R275W) were predicted to be likely pathogenic with CADD and REVEL scores above the selected threshold. However, we noted the occurence of heterozygous p.R256C in three controls (age 80, 81 and 85 years) and one PD patient (AAO = 52 years), which is classified as ‘probably pathogenic’ according to the MDSgenes pathogenicity score. For *PINK1* we found no pathogenic variant classified in ClinVar. However, p.R279H, p.A339T and p.L369P are ‘probably pathogenic’ according to the MDSgene pathogenicity scores, but only when homozygous. Two of these variants (p.R279H, p.L369P) and p.M318L were classified as ‘likely pathogenic’ based on CADD and REVEL scores. In addition, the *PARK7* p.A104S and *ATP13A2* p.R172H, p.S277C, p.P358L, p.R924H and p.R980H heterozygous variants had higher CADD and REVEL scores but were not reported to be pathogenic for PD in ClinVar or MDSgene.

Overall, we described nine pathogenic variants from databases of clinical interest in *LRRK2, PRKN* and *PINK1* in a total of 26 samples (13 PD, 1 PSP and 12 controls, all heterozygous) representing 1.7% of the total cohort (Table 3). Given the zygosity of the variants, only variants in *LRRK2* can be responsible for the disease. AP patients were heterozygous carriers of probably benign variants in *LRRK2* and *PINK1* and a pathogenic variant in *PRKN*.

An extensive screening of *GBA1* variants was previously performed by our team (Pachchek et al., 2023) using *GBA1*-targeted PacBio sequencing in individuals from the Luxembourg Parkinson’s study (660 PD patients, 100 patients with other forms of parkinsonism and 808 controls). We identified 21 rare *GBA1* variants (20 missense and one splice site) in 37 PD patients and 16 controls (representing 5.6% of PD patients and 1.9% controls), which were validated by Sanger sequencing. Eleven rare variants were classified as pathogenic while the others were classified as variants of unknown significance (VUS). For the samples that were both genotyped and screened by targeted *GBA1*-sequencing, we found that none of carriers of rare variants in the studied PD-causing genes, identified within the NeuroChip, harbored an additional pathogenic *GBA1* variant.

### Rare copy number variants in PD-related genes

We initially detected 25,299 CNVs, including 13,862 duplications and 11,437 deletions in 728 controls and 757 PD cases. After all QC and filtering steps, the final number of CNVs was 1,079 CNVs, including 737 duplications and 342 deletions in 373 controls and 366 cases. CNV analysis showed that almost half of the samples (49.7%) carried at least one QC-passed CNV. The length of the CNVs in the entire cohort ranged from 20 kb to 3.0 megabases (Mb) with a median size of 160 kb. The characteristics of our CNV analysis are shown in Table 4.

**Table 4:** Summary of CNV calls from the Luxembourg Parkinson’s study.

	Controls	PD	PSP	LBD	MSAP	CBS	FTDP
Number of samples	728	656	51	27	13	9	1
CNV carriers (n, %)	373 (51.2%)	322 (49.0%)	21 (41.1%)	15 (55.5%)	4 (30.8%)	4 (44.4%)	0 (0%)
Number of CNVs	544	480	25	18	7	5	0
Duplication	377	323	17	12	6	2	0
Deletions	167	157	8	6	1	3	0
CNVs per sample (Mean, SD)	1.8 (0.9)	1.8 (0.9)	1.2 (0.4)	1.3 (0.7)	2.1 (0.9)	1.4 (0.5)	0
Mean size of CNVs (Kb, Mean, SD)	284 (343)	283 (335)	338 (435)	307 (326)	225 (143)	260 (348)	0
Number of SNPs per CNV (Mean, SD)	51.8 (61.6)	55.2 (118)	51.8 (61.6)	55.6 (57.6)	32.3 (12.8)	36.8 (15.6)	0
Filtered out CNVs	11,875	10,598	1,069	350	230	95	3
Filtered out duplication	6,472	5,896	462	95	136	62	2
Filtered out deletions	5,403	4,702	607	255	94	33	1

Details of CNVs called following QC steps in cases and controls. CNV, Copy number variants; PD, Parkinson’s disease; PSP, progressive supranuclear palsy; LBD, Lewy body dementia; MSA, Multiple System Atrophy; CBS, corticobasal syndrome; FTDP, Fronto-temporal dementia with parkinsonism; Kb, Kilobases.

We then explored CNVs overlapping known PD genes and identified 15 CNVs in 18 samples (six controls and 12 PD cases) that were exclusively in the *PRKN* gene (Table 5). None of the *PRKN* CNV carriers had a rare variant in the same gene. We tested the presence of five CNVs by MLPA. As MLPA only covers exonic regions of *PRKN*, three MLPA results were consistent with PennCNV results (Table 5). One duplication was located in exon 2 rather than in a nearby intronic region and one duplication was found to be homozygous covering exon1 rather than heterozygous covering exon 2 (Table 5). After MLPA validation, of the 15 *PRKN* CNVs, eight were single copy deletions, six were single copy duplications and only one was a probably pathogenic homozygous duplication in a late-onset PD patient (AAO = 69 years). Among the PD cases, three *PRKN* heterozygous CNV carriers had an AAO ≤ 50 years (including one patient diagnosed with a juvenile form of PD at the age of 18). One CNV was detected in four samples, while the others were detected in only a single sample (Table 5). Eleven CNVs were considered as rare, since they were not reported in DGV and were spanning structural variants reported in European descent gnomAD_SV dataset with a frequency of less than 1% (Table 5). No clear clinical impact was observed for all the *PRKN* CNVs (uncertain significance in CNV-ClinViewer).

**Table 5:** CNVs detected in any of the PD genes in the Luxembourg Parkinson’s study.

CNV	Length (base)	n SNPs	CN	Gene	CNV region in *PRKN*	DGV Freq	n spanning gnomAD_SV	gnomAD_SV Freq	n Sample	Diagnostic	AAA/AAO	MLPA	MLPA results
chr6:162724247–162855426	131,179	30	3	*PRKN*	intronic (exon 2 – exon 3)	0	2	9.20E-05	4	Control	60	no	*NA*
										Control	50	no	*NA*
										Control	53	no	*NA*
										PD	39	yes	exon 2 one copy duplication
chr6:161692286–161772949	80,663	30	3	*PRKN*	exon 12	0	0	0	1	Control	69	no	*NA*
chr6:162199157–162875706	676,549	153	1	*PRKN*	exon 2–7	0	73	0.14	1	PD	61	no	*NA*
chr6:162279763–162406957	127,195	37	1	*PRKN*	exon 6	0	4	0.01	1	PD	62	no	*NA*
chr6:162305402–162674093	368,691	90	1	*PRKN*	exon 4–6	0	22	0.14	1	Control	35	no	*NA*
chr6:162445941–162513967	68,026	21	1	*PRKN*	exon 5	0	2	0.006	1	PD	63	no	*NA*
chr6:162541706–162750426	208,720	55	1	*PRKN*	exon 3–4	0	23	0.14	1	PD	65	no	*NA*
chr6:162646892–163007394	360,502	91	3	*PRKN*	exon 2–3	0	10	0.001	1	PD	66	no	*NA*
chr6:162653609–163029097	375,488	60	3	*PRKN*	exon 2–3	0	11	0.001	1	PD	18	yes	exon1-4 one copy duplication
chr6:162664364–162750426	86,062	29	1	*PRKN*	exon 3	0	9	0.008	1	PD	56	no	*NA*
chr6:162724247–162889975	165,728	57	1	*PRKN*	exon 2	0	27	0.008	1	PD	75	no	*NA*
chr6:162736336–163054293	317,957	58	3	*PRKN*	exon 2	0	10	0.001	1	Control	64	no	*NA*
chr6:162744935–162914986	170,051	52	3	*PRKN*	exon 2	0	4	0.001	1	PD	69	yes	exon 1 two copies duplication
chr6:162747573–162855426	107,853	22	1	*PRKN*	exon 2	0	15	0.001	1	PD	42	yes	exon 2 one copy deletion
chr6:162945539–163176151	230,612	29	3	*PRKN, PACRG*	exon 1	0	2	4.00E-04	1	PD	52	yes	exon 1 one copy duplication

CN, Copy Number; CN = 1, one copy deletion; CN = 3, one copy duplication. DGV frequency: the number of individuals carrying overlapping CNVs in DGV (Database of Genomic Variants). N spanning gnomAD_SV: number of samples carrying complete overlapping CNVs with the same copy number in gnomAD-SV (the genome aggregation database-structural variants). gnomAD_SV Freq: the highest frequency of the complete overlapping CNVs. AAA (Age at assessment for healthy controls) AAO (Age at Onset for PD cases). MLPA Multiplex ligation-dependent probe amplification.

### Rare digenic variants

Eight individuals (five PD cases, one with PSP and two controls) carried two variants in two different PD-related genes (Table 3). The AAO of the patients ranged from 52 to 71 (mean = 64.3). In particular, in autosomal recessive PD genes, pathogenic *PRKN* p.R256C and *PINK1* p.A339T (in heterozygous state) were detected in the same individual with another probably benign variant. One PD patient (AAO = 62 years) carried the heterozygous *PRKN* deletion (chr6:162,279,763–162,406,957) and also the benign *LRRK2* variant p.R1514Q. Moreover, two controls were carriers of two variants in *PRKN*-*ATP13A2* (81 years old) and in *PRKN*-*PINK1* (70 years old) respectively (Table 3).

### Combining rare single nucleotide and copy number variants in *PRKN*

The number of heterozygous rare pathogenic *PRKN* SNVs (p.Q34fs, p.R256C and p.R275W) was not significantly different between controls (*n* = 6, 0.82%) and PD cases (*n* = 4, 0.5%, value of *p* = 0.6). If we consider all the rare heterozygous CNV deletions as pathogenic loss-of-function variations together with the homozygous duplication, we counted seven PD cases each carrying one rare pathogenic deletion. Overall, the number of heterozygous pathogenic SNVs and CNVs was slightly higher in PD (*n* = 11, 1.64%) than in controls (*n* = 6, 0.82%), but the difference is still not significant (value of *p* = 0.16). The sample size is too small to examine a significant burden of these rare variants on PD risk.

### *MAPT* haplotypes association

We found a statistically significant over-representation of the *MAPT* H1 haplotype in PD (value of *p* = 0.018) and PSP (value of *p* = 0.008) cases, present in 80.5% of PD, 88.0% of PSP cases compared to 76.8% of controls (Supplementary Table 1). No significant association was found between sub-haplotype H1c and any of the investigated diseases (Supplementary Table 1).

### Polygenic risk scores

**Figure 1:**
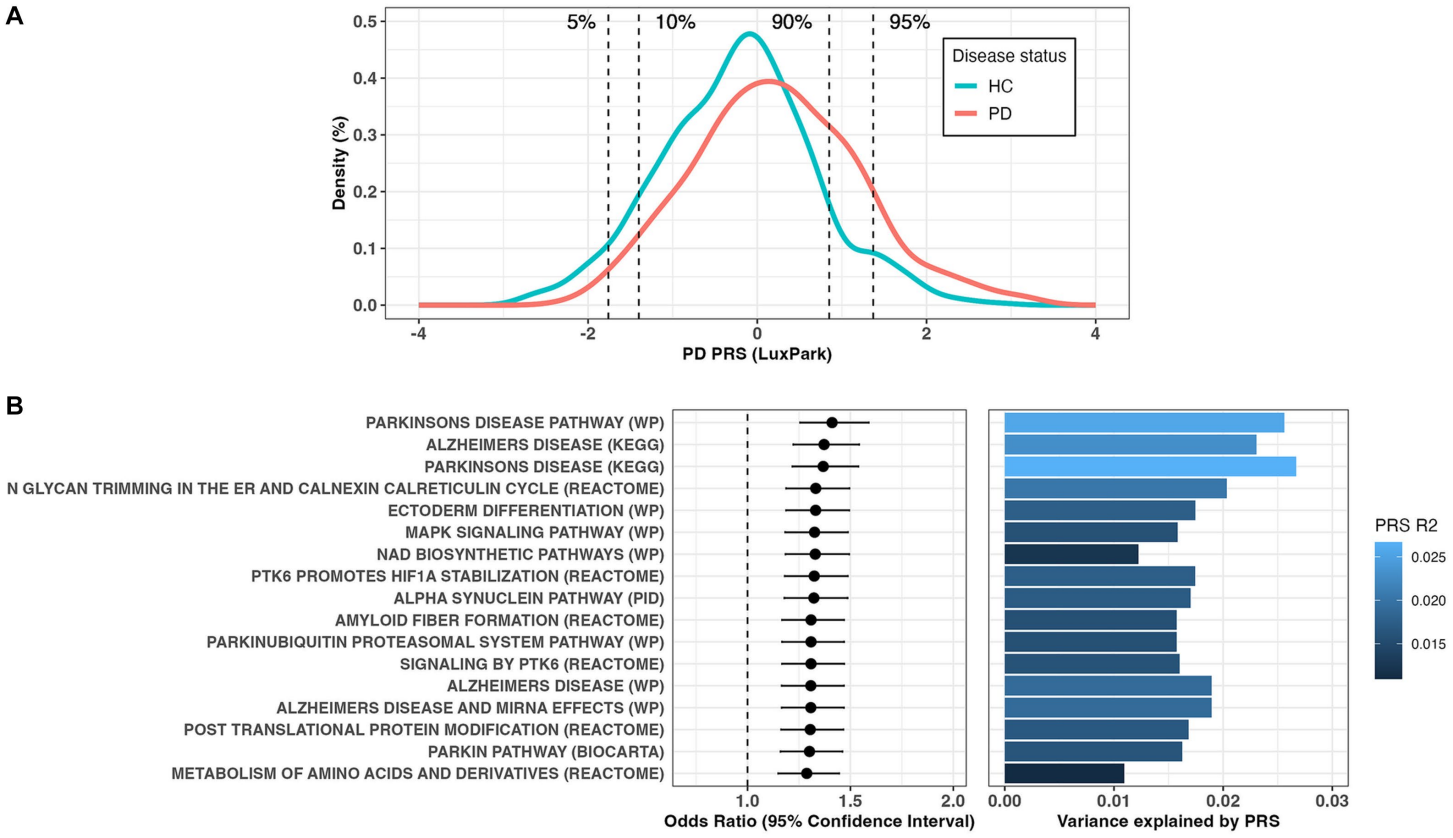
**(A)** Distribution of the polygenic risk score (PRS) between Parkinson’s disease (PD) patients and controls. **(B)** Forest plots showing PRS odds ratio (OR) and 95% confidence interval for the significant canonical pathways associated with PD risk (left panel) and the estimation of variance explained by PRS (right panel).

Using significant common SNVs from the largest PD GWAS summary statistics (Nalls et al., 2019), we calculated the PRS in the Luxembourg Parkinson’s study for 724 controls and 667 PD patients. The PRS model was calculated based on 75 clumped SNPs that showed the best prediction at the GWAS value of *p* threshold of 5e-08 and an observed phenotypic variance R2 of 5.3% (1.9% after adjustment for PD prevalence of 5e-03, empirical value of *p* = 9.9e-05) with an AUC of 62.8%. We found a significant association of PRS with higher PD risk (OR = 1.70[1.50–1.93], *p* = 5.9e-17). The distribution of PRS scores in PD cases and healthy controls was significantly different (Wilcoxon test value of *p* <2.2e-16, Figure 1A). Individuals with the 5 and 10% of highest PRS values had a 9.5-fold [3.9–26.3] (*p* = 1.4e-08) and 5.6-fold [3.3–9.7] (*p* = 1.8e-12) increased risk, respectively, compared to individuals with the lowest 5 and 10% PRS values (Figure 1A). Out of the 3,090 canonical pathways gene-sets representing the most important biological processes and diseases, 17 gene-sets were significantly associated with PD risk (Bonferroni adjusted value of *p* <0.05, Figure 1B; Supplementary Table 2). Among the enriched pathways, the majority were associated with PD (showing the highest R2 values, Figure 1B) and PD pathogenesis (Alpha synuclein, Parkin and ubiquitination related pathways), Alzheimer disease (AD), signal transduction and metabolism of proteins. No gene-set remained significant after excluding the 90 PD GWAS hits region (1 Mb upstream and downstream each locus), indicating the absence of other risk loci acting independently of the known ones.

## Discussion

The current report is a comprehensive genetic description of participants recruited within the monocentric case–control Luxembourg Parkinson’s study, including patients with PD and atypical parkinsonism, with previously described recruitment design and clinical characteristics (Hipp et al., 2018; Pavelka et al., 2022). Previous long-read sequencing of *GBA1* gene in our cohort revealed that 12.1% of PD patients carried *GBA1* variants (Pachchek et al., 2023). Analyzing now the complete Neurochip genotyping data, we investigated the potential effect of rare variants, common low-risk variants and CNVs on the PD pathogenesis. Our findings are consistent with those previously reported in European ancestry datasets.

In the *LRRK2* gene, two well-established pathogenic SNVs were found in five PD patients and two controls with a frequency similar to previous European ancestry datasets (Correia Guedes et al., 2010; Shu et al., 2019). Pathogenic and probably pathogenic variants in the *ATP13A2*, *PARK7*, *PRKN* and *PINK1* genes associated with autosomal recessive PD were found in the heterozygous state, except in one PD patient. The latter carried a homozygous pathogenic *PINK1* variant (p.L369P) and had an AAO of 32 years (Arena and Valente, 2017) reported a similar finding, where homozygous variants in *PINK1* associated with early-onset PD (EOPD) were present in the patient before the age of 45 years. In our study, the number of heterozygous SNVs in the recessive *PARK7*, *ATP13A2, PRKN* and *PINK1* genes was not significantly different between PD cases and controls. Controls carrying these variants were over 50 years of age. The majority had no family history of PD and most of PD patients were not of young onset (AAO > 50 years). Patients with AP carried probably benign heterozygous variants, mainly in *LRRK2* and *PINK1*. Pathogenic *LRRK2* variants have been described in patients with primary tauopathies, although at a low frequency (Wen et al., 2021). In particular, *LRRK2* has recently emerged as a genetic risk factor associated with PSP progression (Jabbari et al., 2021).

We called CNVs from the genotyping data of individuals in the Luxembourg Parkinson’s study and after a stringent quality control and filtering steps, we screened for CNVs overlapping PD causal genes. We identified 12 PD patients who carried CNVs exclusively in the *PRKN* gene, of which three CNVs were validated by MLPA and were reported in patients having a disease AAO ≤ 50 years. Especially, we described a heterozygous exon1-4 duplication in a patient with EOPD (AAO of 18 years) who did not present any rare variant in the PD-related genes studied here. Moreover, we validated by MLPA a homozygous duplication of *PRKN* exon1 in another PD patient with a late disease-onset (69 years). Duplications of *PRKN* exons were previously reported as ‘likely pathogenic’ (Schüle et al., 2015). Indeed, both homozygous and compound heterozygous *PRKN* deletions and duplications have previously been associated with early-onset and familial forms of PD (Elfferich et al., 2011; Kim et al., 2012; Huttenlocher et al., 2015; Ahmad et al., 2023). This was recently reproduced in a large CNV study of 4,800 clinical exome sequencing reports (Pennings et al., 2023). In a Latin American PD cohort, CNVs in *PRKN* were significantly associated with disease progression, with a prevalence of 5.6% in EOPD cases (Sarihan et al., 2021).

We found that six PD cases and one PSP case carried digenic variants in two different PD-related genes (*LRRK2-PRKN*, *LRRK2-PINK1, PINK1*-*ATP13A2*) with AAO greater than 50 years. Hitherto, only a few studies have identified digenic variants of PD-related genes [*LRRK2-PRKN* (Dächsel et al., 2006), *PINK1*-*PARK7* (Tang et al., 2006) or *PRKN-PINK1* (Hayashida et al., 2021)]. Previous studies reported that carriers of digenic variants in *PRKN* and *PINK1* develop the disease at a younger age and exhibit distinctive symptoms such as schizophrenia, facial dyskinesia, grimacing and severe dysarthria (Funayama et al., 2008) and also epilepsy and essential tremor (Hayashida et al., 2021). However, the digenic variants reported in this study differ from those previously described, and carriers of these variants have an older age at onset. Nonetheless, the ambiguity surrounding digenic variants persists due to the limited number of reported cases. A detailed familial and clinical study could be carried for every individual, to confirm that the combination of these heterozygous variants, in the context of a digenic inheritance, may point out the phenotype observed in PD and PSP cases.

In our study, we did not find a significant overrepresentation of rare heterozygous SNVs and CNVs in *PRKN*. In particular, heterozygous pathogenic *PRKN* variants were not significantly more frequent in controls than in PD cases. Homozygous or compound heterozygous variants in this gene were the most common cause of EOPD (Kilarski et al., 2012), while heterozygous loss of *PRKN* function may be a potential risk factor for developing PD (Klein et al., 2007; Huttenlocher et al., 2015; Castelo Rueda et al., 2021; Lubbe et al., 2021) and therefore identifying individuals at increased risk might be useful in the prodromal phase. However, this role of heterozygous *PRKN* is still under debate, as previous reports suggested a lack of association with PD (Kay et al., 2010). Recently, in a larger association study, Yu and colleagues fully sequenced *PRKN* in a PD cases/controls cohort from European ancestry, including 1965 late-onset and 553 early-onset, and concluded that heterozygous SNVs or CNVs in *PRKN* are not associated with EOPD (Yu et al., 2021). They reported that 1.52% of PD and 1.8% of controls were carriers. Here, using a SNP array based on CNVs and SNPs screening, we showed similar percentages (1.64% of PD and 0.82% of controls) with non-significant differences between controls and mainly late-onset PD cases.

Potential neuroprotective PD therapies and clinical trials are now targeting specific PD subtypes based on genetic markers causing or increasing the disease risk, such as therapies targeting *LRRK2*, *GBA1* and alpha-synuclein (Sardi et al., 2018). Parkin-proved disease is characterized by a slow motor progression, preserved cognition and a limited increase in dopaminergic medication over time (Menon et al., 2023). Moreover, severe loss of dopaminergic neurons was observed in homozygous *PRKN* carriers without Lewy bodies formation, which is one of the major markers of idiopathic PD (Mata, 2004). Confirming the potential role of heterozygous *PRKN* variants in the pathogenesis of PD will be crucial, despite the lack of data describing PD conversion of individuals carrying these genetic risk factors.

Beyond the effects of rare variants, we have demonstrated that individuals carrying the *MAPT* H1 haplotype are at higher risk to develop PD and PSP. These findings are consistent with previous studies that have assessed the H1 haplotype as a PD (Zabetian et al., 2007) and PSP (Baker et al., 1999) risk factor. We did not detect the association of PD or any forms of atypical Parkinsonism with H1c sub-haplotype which was strongly associated with risk for PSP and CBD (Myers et al., 2005; Kouri et al., 2015) but not PD (Zabetian et al., 2007). Next, we estimated the total cumulative contribution of common low-risk SNVs by calculating the PRS. Our PRS model of disease risk showed an expected trend similar to previous reports showing that PRS discriminates PD cases from unaffected individuals (Dehestani et al., 2021). Several polygenic analyses have become standard tools for assessing the risk for complex disorders and an accurate method for predicting disease status and identifying high-risk individuals (Lewis and Vassos, 2020). Next, we looked up at how thousands of biological pathways might contribute to the risk of developing PD. In addition to pathways already associated with PD and AD, molecular processes underlying proteins metabolism, signal transduction and post-translational protein modification were among the most important contributors to PD risk. The metabolic dysfunction, energy failure and redox imbalance observed in PD were considered obvious features to qualify PD as a complex metabolic disorder (Anandhan et al., 2017). In addition, disruption of any stage in the protein life cycle could engender PD pathology (Langston and Cookson, 2020). Comparing our results with a previous large-scale gene set-specific PRS studies that reported the involvement of multiple processes in the etiology of PD (Bandres-Ciga et al., 2020), similar molecular processes were found here. However, other processes such as immune response, synaptic transmission and endosomal-lysosomal dysfunction were not highlighted which may be due to the smaller sample size in our dataset. Pathway PRSs are expected to provide important insights into the complex heterogeneity of PD and how patients respond to treatment, by generating biologically traceable therapeutic targets from polygenic signals (Choi et al., 2023). We are aware that our study has several limitations: (1) the sample size was not large enough to have sufficient statistical power to perform further analysis, such as GWAS for PD risk or AAO, genome-wide CNV burden or human leukocyte antigen (HLA) association; (2) not all known variants associated with PD can be accurately assessed by the NeuroChip and we might have missed some mutated alleles, even though we confirmed all the identified variants by Sanger sequencing; (3) we used best practices to call CNVs from genotyping data (Sarihan et al., 2021), and thus we will always miss small CNVs that are systematically filtered out. Moreover, we could validate only few of the called CNVs with MLPA and (4) our analysis revealed a higher incidence of first-degree family history among controls. Therefore, caution must be exercised when searching for recessive disease forms. Although proxy cases have proven their effectiveness in large scale study investigating common variants (Nalls et al., 2019) and have also highlighted their usefulness in detecting rare variants (Makarious et al., 2023).

## Conclusion

In conclusion, our study has successfully performed a comprehensive genetic baseline characterization of the Luxembourgish PD case–control cohort, investigating rare variants, CNVs and PRSs. Our findings do not support an association between PD risk and rare heterozygous *PRKN* variants. We also described a possible role of *LRRK2* in AP and new possible digenic inheritance patterns in PD. Together with other studies in different European populations, our findings will advance the understanding of PD pathogenesis and genetics and could redefine the development of future therapeutic targets and therapies.

## Data availability statement

Genetic and clinical data for this manuscript are not publicly available as they are linked to the internal regulations of the Luxembourg Parkinson’s Study. Requests for accessing the datasets can be directed to request ncer-pd@uni.lu. The code behind the analyses is available under Apache-2.0 license. More information on how to request access to the data and the code is available at https://doi.org/10.17881/ea65-t027.

## Ethics statement

The studies involving humans were approved by the National Research Ethics Committee (CNER Ref: 201407/13). The studies were conducted in accordance with the local legislation and institutional requirements. The participants provided their written informed consent to participate in this study.

## NCER-PD consortium member

Geeta Acharya, Gloria Aguayo, Myriam Alexandre, Wim Ammerlann, Katy Beaumont, Camille Bellora, Jessica Calmes, Lorieza Castillo, Gessica Contesotto, Daniela Esteves, Guy Fagherazzi, Jean-Yves Ferrand, Manon Gantenbein, Marijus Giraitis, Jérôme Graas, Gaël Hammot, Anne-Marie Hanff, Estelle Henry, Michael Heymann, Alexander Hundt, Sonja Jónsdóttir, Jochen Klucken, Rejko Krüger, Pauline Lambert, Victoria Lorentz, Paula Cristina Lupu, Guilherme Marques, Deborah Mcintyre, Chouaib Mediouni, Myriam Menster, Maura Minelli, Ulf Nehrbass, Fozia Noor, Magali Perquin, Rosalina Ramos Lima, Eduardo Rosales, Estelle Sandt, Margaux Schmitt, Amir Sharify, Kate Sokolowska, Hermann Thien, Johanna Trouet, Olena Tsurkalenko, Michel Vaillant, Mesele Valenti, Luxembourg Institute of Health, Strassen, Luxembourg; Muhammad Ali, Giuseppe Arena, Rudi Balling, Michele Bassis, Regina Becker, Ibrahim Boussaad, Kathrin Brockmann, Piotr Gawron, Soumyabrata Ghosh, Enrico Glaab, Elisa Gómez De Lope, Valentin Groues, Anne Grünewald, Wei Gu, Linda Hansen, Michael Heneka, Sascha Herzinger, Jacek Jaroslaw Lebioda, Yohan Jaroz, Quentin Klopfenstein, Jochen Klucken, Rejko Krüger, Zied Landoulsi, Tainá M. Marques, Patricia Martins Conde, Patrick May, Francoise Meisch, Michel Mittelbronn, Michel Mittelbronn, Sarah Nickels, Marek Ostaszewski, Clarissa P. C. Gomes, Sinthuja Pachchek, Claire Pauly, Laure Pauly, Lukas Pavelka, Armin Rauschenberger, Rajesh Rawal, Dheeraj Reddy Bobbili, Kirsten Roomp, Isabel Rosety, Stefano Sapienza, Venkata Satagopam, Sabine Schmitz, Reinhard Schneider, Jens Schwamborn, Ekaterina Soboleva, Rebecca Ting Jiin Loo, Christophe Trefois, Carlos Vega, Maharshi Vyas, Paul Wilmes, Evi Wollscheid-Lengeling, Luxembourg Centre for Systems Biomedicine, University of Luxembourg, Esch-sur-Alzette, Luxembourg; Guy Berchem, Nico Diederich, Marijus Giraitis, Linda Hansen, Jochen Klucken, Rejko Krüger, Laura Longhino, Romain Nati, Beatrice Nicolai, Claire Pauly, Lukas Pavelka, Elodie Thiry, Liliana Vilas Boas, Gelani Zelimkhanov, Centre Hospitalier de Luxembourg, Strassen, Luxembourg; Michel Mittelbronn, Friedrich Mühlschlegel, Laboratoire National de Santé, Dudelange, Luxembourg; Alexandre Bisdorff, Rene Dondelinger, Centre Hospitalier Emile Mayrisch, Esch-sur-Alzette, Luxembourg; Sylvia Herbrink, Centre Hospitalier du Nord, Ettelbrück, Luxembourg; Roseline Lentz, Parkinson Luxembourg Association, Leudelange, Luxembourg; Michele Hu, Oxford Parkinson’s Disease Centre, Nuffield Department of Clinical Neurosciences, University of Oxford, Oxford, United Kingdom; Richard Wade-Martins, Oxford Parkinson’s Disease Centre, Department of Physiology, Anatomy and Genetics, University of Oxford, Oxford, United Kingdom; Clare Mackay, Oxford Centre for Human Brain Activity, Wellcome Centre for Integrative Neuroimaging, Department of Psychiatry, University of Oxford, Oxford, United Kingdom; Daniela Berg, Kathrin Brockmann, Thomas Gasser, Inga Liepelt, Center of Neurology and Hertie Institute for Clinical Brain Research, Department of Neurodegenerative Diseases, University Hospital Tübingen, Tübingen, Germany; Brit Mollenhauer, Paracelsus-Elena-Klinik, Kassel, Germany; Katrin Marcus, Ruhr-University of Bochum, Bochum, Germany; Robert Liszka, Westpfalz-Klinikum GmbH, Kaiserslautern, Germany; Walter Maetzler, Department of Neurology, University Medical Center Schleswig-Holstein, Kiel, Germany; Mariella Graziano, Association of Physiotherapists in Parkinson’s Disease Europe, Esch-sur-Alzette, Luxembourg; Nadine Jacoby, Private Practice, Ettelbruck, Luxembourg; Jean-Paul Nicolay, Private Practice, Luxembourg, Luxembourg.

## Author contributions

ZL: Conceptualization, Data curation, Investigation, Methodology, Writing – original draft. SP: Conceptualization, Data curation, Investigation, Methodology, Writing – review & editing. DB: Data curation, Writing – review & editing. LP: Data curation, Writing – review & editing. PM: Conceptualization, Supervision, Writing – review & editing, Investigation, Methodology. RK: Conceptualization, Supervision, Writing – review & editing, Investigation, Methodology.

## Footnotes

^1^ https://www.mdsgene.org

^2^ https://www.gsea-msigdb.org/
