# Supplementary material for: Genetic landscape of Parkinson’s disease and related diseases in Luxembourg
Source: Front Aging Neurosci. 2023 Dec 20;15:1282174. doi: 10.3389/fnagi.2023.1282174 (PMC10761438; doi:10.3389/fnagi.2023.1282174)
Supplement: Supplementary file 1 [file Data_Sheet_1.docx]

Supplementary Material

Parkinson's Genetics Study in Luxembourg

Zied Landoulsi ^1†^; Sinthuja Pachchek Peiris ^1†^; Dheeraj Reddy Bobbili ^1^, Lukas Pavelka^2,3^, Patrick May^1*^, and Rejko Krüger ^1,2,3,*^ on behalf of the NCER-PD Consortium

*** Correspondence:**Patrick May: patrick.may@uni.lu, Rejko Krüger: rejko.krueger@uni.lu

## Supplementary Figures:


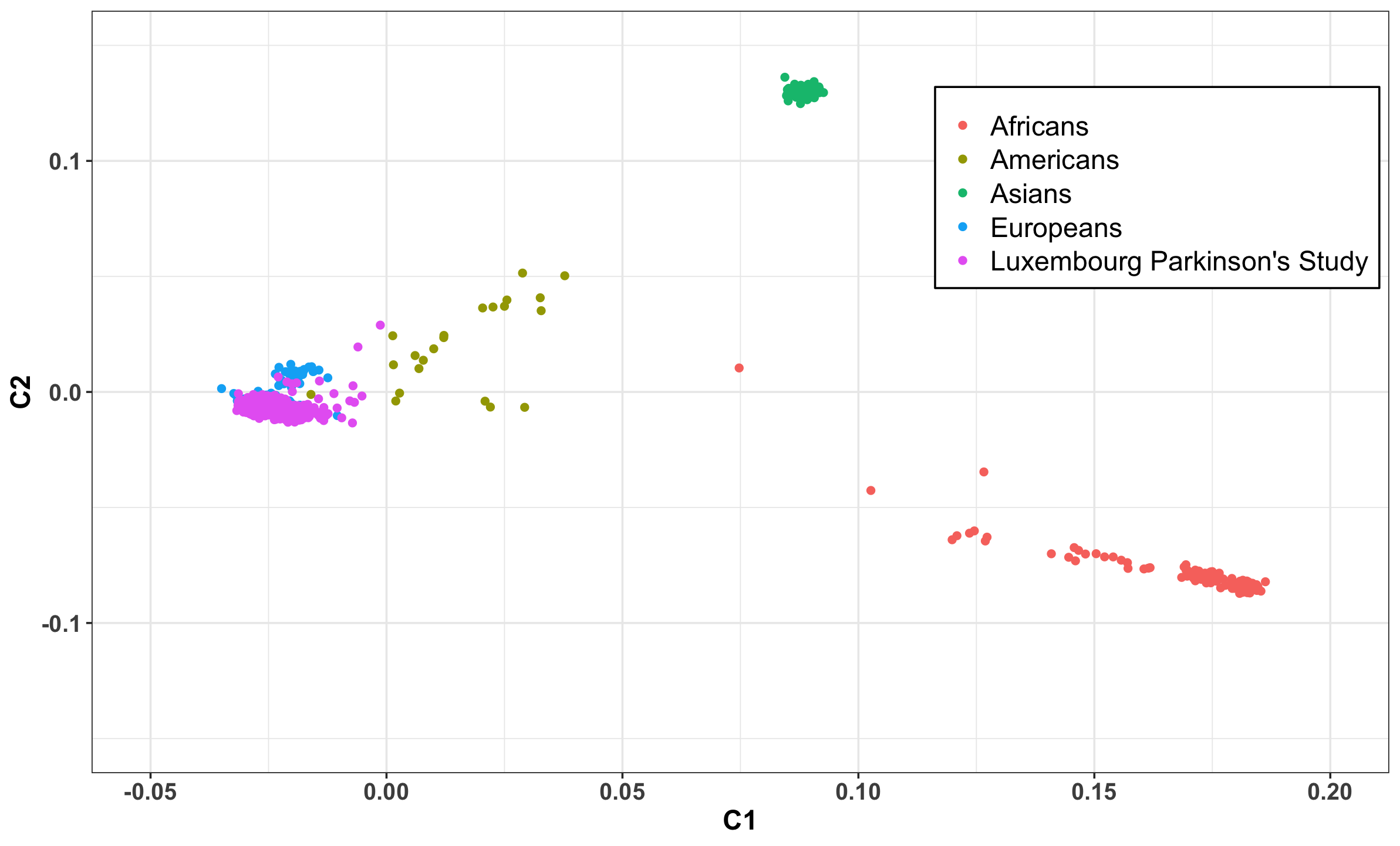


**Supplementary Figure 1.** PCAs of the Luxembourg Parkinson’s Study individuals (in purple) and the 1000 Genomes population for comparison (color-coded by different populations).

**Supplementary Table 1: Association of *MAPT* H1/H2 haplotypes and H1c sub-haplotype with PD and atypical Parkinsonism risk.** Genotypic and allelic frequency distribution of the SNP rs1800547 tagging the H1/H2 haplotype and the sub-haplotype H1c.

|  | **H1/H1** | **H1/H2** | **H2/H2** | **chi-squared test p-value** | **H1 frequency** | **Allelic OR [95 CI]** | **Fisher's test p-value** | **H1c/H1c** | **H1c/H2** | **H1c frequency** | **Allelic OR [95 CI]** | **Fisher's test p-value** |
| --- | --- | --- | --- | --- | --- | --- | --- | --- | --- | --- | --- | --- |
| **Controls** | 432 | 249 | 43 |  | 0.76 |  |  | 20 | 40 | 0.05 |  |  |
| **PD** | 436 | 203 | 28 | 0.062 | 0.8 | 1.24 [1.03-1.50] | **0.018** | 15 | 29 | 0.04 | 0.79 [0.55-1.13] | 0.192 |
| **PSP** | 39 | 10 | 1 | 0.036 | 0.88 | 2.20 [1.18-4.48] | **0.008** | 1 | 2 | 0.04 | 0.71 [0.19-1.96] | 0.651 |
| **LBD** | 9 | 15 | 1 | 0.033 | 0.66 | 0.58 [0.31-1.13] | 0.088 | 1 | 0 | 0.04 | 0.71 [0.08-2.81] | 1 |
| **MSA** | 7 | 5 | 1 | 0.717 | 0.73 | 0.81 [0.32-2.32] | 0.641 | 0 | 2 | 0.07 | 1.42 [0.16-5.91] | 0.652 |
| **CBS** | 6 | 3 | 1 | 0.736 | 0.75 | 0.91 [0.30-3.19] | 0.793 | 0 | 1 | 0.05 | 0.9 [0.02-5.81] | 1 |
| **FTD-P** | 0 | 1 | 0 | *NA* | *NA* | *NA* | *NA* | 0 | 0 | 0 | *NA* | *NA* |

**Supplementary Table 2: Canonical pathways significantly associated with PD risk in the Luxembourg Parkinson’s study.** PRS R2: variance explained by polygenic risk score, SE: standard error, P-value (after Bonferroni correction), N SNP: Number of SNPs included in each gene-set analysis

| **Gene set** | **N genes** | **Beta** | **SE** | **P-value** | **PRS R2** | **N SNPs** |
| --- | --- | --- | --- | --- | --- | --- |
| PARKINSONS DISEASE PATHWAY (WP) | 71 | 0.344278 | 0.060147 | 3.10E-05 | 0.025608 | 16 |
| ALZHEIMERS DISEASE (KEGG) | 166 | 0.316265 | 0.059383 | 3.10E-04 | 0.023056 | 11 |
| PARKINSONS DISEASE (KEGG) | 129 | 0.313221 | 0.059654 | 4.60E-04 | 0.026692 | 42 |
| N GLYCAN TRIMMING IN THE ER AND CALNEXIN CALRETICULIN  CYCLE (REACTOME) | 34 | 0.286283 | 0.058815 | 3.40E-03 | 0.020331 | 356 |
| ECTODERM DIFFERENTIATION (WP) | 139 | 0.285898 | 0.05872 | 3.40E-03 | 0.017453 | 5 |
| MAPK SIGNALING PATHWAY (WP) | 244 | 0.282071 | 0.058492 | 4.30E-03 | 0.015836 | 33 |
| NAD BIOSYNTHETIC PATHWAYS (WP) | 22 | 0.284523 | 0.059372 | 4.90E-03 | 0.012237 | 9 |
| PTK6 PROMOTES HIF1A STABILIZATION (REACTOME) | 6 | 0.280816 | 0.058907 | 5.90E-03 | 0.017434 | 7 |
| ALPHA SYNUCLEIN PATHWAY (PID) | 31 | 0.279482 | 0.05915 | 7.10E-03 | 0.017014 | 17 |
| AMYLOID FIBER FORMATION (REACTOME) | 105 | 0.269046 | 0.058621 | 1.40E-02 | 0.015743 | 4 |
| PARKIN-UBIQUITIN PROTEASOMAL SYSTEM PATHWAY (WP) | 71 | 0.269046 | 0.058621 | 1.40E-02 | 0.015743 | 4 |
| SIGNALING BY PTK6 (REACTOME) | 53 | 0.268987 | 0.058748 | 1.50E-02 | 0.016014 | 5 |
| ALZHEIMERS DISEASE (WP) | 261 | 0.268217 | 0.058881 | 1.60E-02 | 0.018947 | 7 |
| ALZHEIMERS DISEASE AND MIRNA EFFECTS (WP) | 320 | 0.268217 | 0.058881 | 1.60E-02 | 0.018947 | 7 |
| POST TRANSLATIONAL PROTEIN MODIFICATION (REACTOME) | 1419 | 0.26604 | 0.059004 | 2.00E-02 | 0.016832 | 66 |
| PARKIN PATHWAY (BIOCARTA) | 8 | 0.263226 | 0.058883 | 2.40E-02 | 0.016246 | 10 |
| METABOLISM OF AMINO ACIDS AND DERIVATIVES (REACTOME) | 366 | 0.252922 | 0.058534 | 4.90E-02 | 0.010947 | 6 |
